# Comparing Intraoperative Ultrasound and Postoperative Computed Tomography in Predicting Chronic Subdural Haemorrhage Clearance – A Pilot Study

**DOI:** 10.21315/mjms-03-2026-190

**Published:** 2026-06-30

**Authors:** Jesse Zen Ngui, Nurfaten Hamzah, Abdul Rahman Izaini Ghani, Zamzuri Idris, Sii Hieng Wong Albert, Jafri Malin Abdullah

**Affiliations:** 1 Department of Neurosurgery, Sarawak General Hospital, 93586 Kuching, Sarawak, Malaysia; 2 Department of Neurosciences and Brain and Behaviour Cluster, Hospital Pakar USM, Universiti Sains Malaysia Health Campus, 16150 Kubang Kerian, Kota Bharu, Kelantan, Malaysia

**Keywords:** chronic subdural, hematoma, ultrasound, computed tomography, recurrence, clearance

## Abstract

**Background:**

Recurrence following chronic subdural hematoma (CSDH) surgery remains a vexing problem. A clinically useful method of accurately predicting recurrence would be of utility in risk-stratifying patients and reduce the morbidity of further surgery. This study is a prospective single armed study comparing the use of intraoperative ultrasound to predict recurrence compared to postoperative computed tomography imaging.

**Methods:**

Data were collected prospectively on patients who had surgery for CSDH in a single centre for over one year. Intraoperative ultrasound was performed prior to and after drainage. Postoperative computed tomography (CT) scans were also obtained for all patients. Demographic and radiologic data, recurrence rate, complication rate and functional outcomes were analysed.

**Results:**

Twenty-five patients were included in the study, with 16 males (64%) and nine females (36%), averaging 69 years of age, with 48% on antiplatelets and/or anticoagulants. All underwent burr-hole and drainage. A 48% clearance rate as determined by ultrasound was achieved, with a 52% rate of hematoma clearance seen on postoperative CT scan. Inpatient mortality was one patient (4%), and four (16%) patients had recurrence. One patient (4%) suffered a postoperative wound infection. Intraoperative ultrasound clearance showed a trend toward reduced recurrence [RR 0.476 (95% CI: 0.304, 0.746)].

**Conclusion:**

Intraoperative ultrasound-determined clearance may be more predictive of clearance than postoperative CT scan for CSDHs. This finding needs to be confirmed with larger studies.

## Introduction

Chronic subdural hematomas (CSDH) are among the most common neurosurgical conditions encountered in clinical practice. Incidence continues to increase due to global demographic shifts resulting in an ageing population, and is projected to become the most common neurosurgical condition in 2030 (1).

The clinical presentation of CSDH is highly heterogeneous based on aetiology, with multiple risk factors identified, most notably head trauma reported in 59% of cases (2) and the use of anticoagulants (3). Current treatment modalities for CSDH include surgical evacuation, middle meningeal artery embolisation, and pharmacotherapy with steroids or antifibrinolytics (4). Despite these interventions, recurrence remains common, with a median rate of 15% reported in the literature (5), contributing to poor functional outcomes. Postoperative imaging using CT or magnetic resonance imaging (MRI) is typically performed to assess hematoma clearance. Radiologic markers such as persistent midline shift, internal hematoma architecture, and residual acute components have been associated with recurrence (6); however, their clinical predictive value remains debated (7). Ongoing research aimed at identifying reliable biomarkers and effective preventive strategies continues investigations.

Few studies have explored the role of ultrasound in evaluating CSDH. This study aims to compare the performance of intraoperative ultrasound examination with that of standard postoperative CT in predicting CSDH clearance.

## Methods

This study is a single-centre, single-arm, prospective pilot study conducted over one year. Patients were recruited using convenience sampling, and formal informed consent was obtained from the patient or their legal next of kin. Inclusion criteria comprised patients aged > 18 years at diagnosis, those diagnosed with CSDH on CT or MRI imaging prior to surgery, and patients scheduled for and undergoing burr hole and hematoma evacuation. Exclusion criteria included patients under 18, pregnant individuals, those with intracranial vascular malformations, and patients not undergoing burr-hole surgery for CSDH, including those treated with craniotomy and clot evacuation. All surgeries and intraoperative ultrasounds were performed by or under the direct supervision of the primary author. A BK Ultrasound FlexFocus 800 system equipped with a burr hole probe (coded 8863; outer diameter 14mm) was used, prepared with a sterile sheath according to manufacturer guidelines. Preoperative demographic, clinical, and radiological data were collected, including hematoma classification according to the Nakaguchi classification (8).

The surgical technique was standardised across all patients. Preoperative correction of coagulopathy with the appropriate medications or blood products was performed. Perioperative antibiotics and antiepileptics were administered. Procedures were performed under general anaesthesia; one or two burr holes (15 mm diameter) were created per hemisphere, depending on preoperative findings. Hematoma drainage was achieved through controlled irrigation with warm saline. Intraoperative ultrasound was performed prior to dural opening and following irrigation and hematoma clearance. CSDH was diagnosed as a hyperechoic layer overlying the cortical surface, as described by Shimizu et al. (9). Ultrasound characteristics were documented and categorised using the Nakaguchi classification. After evacuation, the subdural space was filled with sterile saline, and an ultrasound was repeated. The “starry sky sign” resulting from air bubbles generating hyperechoic signals within a saline collection is taken as complete clearance within the visualised field. Residual hematomas were seen either as an additional hyperechoic layer persisting after drainage or as membranes distinct from the cortical surface after irrigation. Accordingly, ultrasound findings following irrigation were graded as “Cleared” with complete clearance, and “Residual” with remaining collections.

Figures 1 and 2 show examples of CSDH on ultrasound before and after clearance. Figure 3 demonstrates an example in which irrigation failed to achieve complete clearance of a CSDH. The obtained imaging data were saved for review. A subperiosteal drain was used in all cases and kept in place for about two days. The first postoperative CT scan was performed at 24 to 48 hours. Collected findings included residual collection thickness, midline shift, and new acute hematomas; measurements of pneumocranium, if present, were obtained. Pneumocranium was classified as thick if the maximal perpendicular diameter was > 10 mm and thin (< 10 mm) otherwise. Postoperative complications and functional outcomes were recorded. Discharged patients were followed up for at least three months. The primary outcome measured was recurrence, defined as clinical signs and symptoms similar to his initial presentation or suggestive of a CSDH, according to Oh et al. (10). Late postoperative imaging was performed when clinically indicated.

Data were analysed using IBM SPSS Statistics (version 25). Variables are presented as frequencies and percentages. Univariate analysis was performed to evaluate associations between clinical variables and outcomes. All statistical tests were two-sided, and *P*-values < 0.05 were considered statistically significant. Ultrasound data were also reviewed by another rater, and findings were compared using Cohen’s kappa for inter-rater consistency. Given the exploratory design of the study and the limited sample size, results were interpreted with caution.

## Results

### Patients Characteristics

A total of 25 patients who underwent burr hole evacuation for CSDH were included. The mean age was 69.3 ± 8.37 years (range, 53 to 87 years), and 64% were male. A history of recent falls was reported in 56% of patients. Hypertension was the most prevalent comorbidity, affecting 80%, followed by diabetes mellitus at 52% and ischaemic heart disease at 32%. Antiplatelet therapy was administered to 32% of the patients, while 20% received anticoagulants. Most patients presented with a Glasgow Coma Scale (GCS) score of 13 to 15 (68%), whereas 32% had scores between 8 and 12. Table 1 summarises the baseline demographic, clinical, and preoperative radiological characteristics.

### Radiological and Intraoperative Findings

Preoperative CT imaging demonstrated bilateral hematomas in 40% of patients, while 64% showed an acute component. The mean clot thickness was 2.0 cm, with a mean midline shift of 0.67 cm (Table 1). The homogeneous subtype was the most common (31%), followed by trabeculated (26%), separated (23%), and laminated (20%) subtypes according to the Nakaguchi classifications. Intraoperative ultrasound demonstrated complete CSDH clearance (starry sky sign) in 48% of patients, whereas 52% had residual CSDH. Postoperative CT scans demonstrated complete clearance in 13 patients (52%), with residual collections present in 12 patients (48%). Pneumocranium was observed in 14 patients (56%), and 11 cases (44%) were classified as thick (> 1 cm). Inter-rater agreement for ultrasound interpretation was assessed using Cohen’s kappa, demonstrating moderate agreement (κ = 0.54). Table 2 summarises the radiological and ultrasound findings.

### Postoperative Outcomes

The mean postoperative hospital stay was approximately 5.96 days (ranging from 3 to 17 days). Postoperative complications included tension pneumocephalus (4%), new acute subdural haemorrhage (8%), surgical site infection (4%), and systemic complications such as pneumonia (8%) and cardiovascular events (4%). At three-month follow-up, recurrence occurred in four patients (16%). Functional outcomes showed improvement, with mean mRS scores decreasing from 1.80 at discharge to 1.33 at three months. Postoperative outcomes are presented in Table 2. All four patients with clinical recurrence exhibit residual hematomas on ultrasound, while two patients were confirmed to have residual hematomas on postoperative CT imaging. Table 3 summarises the ultrasound and CT findings and their correlation to clinical recurrence.

### Factors Associated with Recurrence

Univariate analysis was conducted to assess the association between clinical, radiologic, and intraoperative variables and recurrence of CSDH. No demographic or clinical variables, including age (*P* = 0.166), gender (*P* = 0.542), diabetes mellitus (*P* = 0.265), hypertension (*P* = 0.166), anticoagulant use (*P* = 0.166), or antiplatelet use (*P* = 0.188), were significantly associated with recurrence.

Similarly, radiological factors such as the presence of an acute component (*P* = 0.159), hematoma thickness exceeding 2 cm (*P* = 0.458), and postoperative pneumocephalus exceeding 1 cm (*P* = 0.108) were not significantly associated with recurrence. Surgical factors, such as the number of burr-holes performed (*P* = 0.532), also showed no significant association with recurrence.

Among the predictors, the odds ratio for ultrasound clearance was 0.476 (95% CI: 0.304, 0.746), indicating that ultrasound clearance is statistically significant for predicting postoperative recurrence. In contrast, postoperative CT imaging clearance was not associated with recurrence (*P* = 0.604; OR 0.750; 95% CI: 0.088, 6.388). The phi coefficient between ultrasound and CT hematoma clearance was 0.368, indicating a moderate correlation between the findings of the two modalities. Table 4 summarises the above findings.

Overall, these results indicate that intraoperative ultrasound assessment of CSDH clearance might have potential prognostic value for predicting recurrence. In contrast, demographic, clinical, and other radiological factors were not predictive in this small pilot cohort.

Formal predictive modelling was not performed due to the very small number of recurrence events (*n* = 4). All findings should be interpreted as exploratory and hypothesis-generating, acknowledging limited statistical power, outcome imbalance, and lack of multivariable adjustment.

## Discussion

The formation of CSDH involves the development and persistence of a neovascularised inflammatory membrane within the subdural space following an initial inciting event, such as minor head injury, with proinflammatory mediators and cytokines driving this process. Recurrent hematomas from fragile capillaries within these membranes contribute to hematoma expansion, inducing neurologic symptoms from mass effect (11). It is hypothesised that removing or attenuating these membranes reduces CSDH recurrence (12). The standard treatment for symptomatic CSDH is surgical evacuation of the clot, typically via burr hole or twist-drill craniotomy, and, if feasible, fenestration or removal of the underlying membranes. Pharmacologic and endovascular modalities to prevent recurrence, such as middle artery embolisation, corticosteroids, and antifibrinolytics, are also used, as summarised by Fakhry et al. (13).

The standard treatment for symptomatic CSDH is a commonly encountered neurosurgical problem, with a reported incidence of up to 20% to 30% in reported cohorts. From a surgical perspective, limited access or visualisation when addressing underlying membrane formations with burr holes or craniostomies may also contribute. Recurrences often require repeat surgery, including the use of more invasive techniques such as craniotomies, which may pose significant morbidity for frail and elderly patients who form a large proportion of CSDH cohorts. A 2025 systematic review by de Rooij et al. (14) concluded that many patients have unsatisfactory functional outcomes following redo surgery for CSDH. Identifying specific clinical markers associated with recurrence would aid in risk stratification of patients and enhance the efficient use of advanced treatments such as middle meningeal artery embolisation.

Sonographic examination of the intracranial region is standard in infants, whose skulls have acoustic windows such as open fontanelles. Differentiation of the different tissue layers, including the cortex proper, arachnoidal, and subdural layers, is possible due to varying echogenic characteristics of these tissues (15). Our study extends this principle to adult patients. Intraoperative ultrasound in cranial surgery has become a useful adjunct for other indications, such as localising deep-seated masses, estimating the extent of lesion resection, localising lesions, and guiding catheter placement, due to its ability to generate real-time images (16). Shimizu et al. (9) presented a technical note that this study has adapted, demonstrating the utility of CSDH drainage and introducing certain sonographic findings adopted in this study. Other studies demonstrating the technical feasibility of ultrasound in identifying CSDH include Niesen et al. (17) and Cattalani et al. (18). This study provides new data indicating that ultrasonographic clearance may be more predictive of clearance than postoperative CT following surgery for CSDH.

In this study, technical feasibility was not a limitation, as adequate intraoperative images were obtained in all patients, suggesting good reproducibility. The equipment was easy to sterilise and prepare for surgical use. Sonographic examination could also be performed rapidly, often completed in less than a minute, allowing repeat evaluations and acquisition of multiple images during surgery. The “starry sky” sign was significantly associated with definitive clearance of CSDH in this study, as evidenced by the odds ratio < 1.0. In contrast, postoperative CT examination markers did not predict hematoma clearance in this cohort. Although prior systematic reviews have identified CT-based markers associated with recurrence, such as larger preoperative clot thickness, bilateral hematomas, postoperative pneumocranium, presence of membranes, inadequate brain to re-expand postoperatively, postoperative persistence of midline shift, and specific hematoma internal architectural subtypes (19). This discrepancy may be attributable to heterogeneity in data collection methods and variations in the definition of recurrence. Furthermore, postoperative subdural collections are normal on CT scan and are expected to take up to a year to resorb fully (20), and if small residual hematomas are present, they may not exert the necessary mass effect to cause symptoms. Furthermore, clinicians frequently consider limited radiologic improvement acceptable if the patient shows good clinical response, rather than opting for repeat surgery, which could introduce reporting bias. Another possible explanation is that intraoperative ultrasound may offer higher local resolution than CT (21), allowing more precise inspection of the subdural space for residual collections or membranes. In this study, subcentimeter structures, such as membranes, were frequently visualised and were not apparent on postoperative CT. In addition, real-time visualisation facilitates intraoperative decision-making, such as determining whether further irrigation is required. The membrane clearance rate in this study was 48%, which is considered low because membrane manipulation was avoided for safety reasons. Shimizu et al. (9) reported a clearance rate of 87.5%, suggesting a potential role of ultrasound in safe performance of this surgical manoeuvre. Despite these improvements, the functional status and recurrence rates in this patient cohort were within reported ranges in recent literature (22). There were no complications with ultrasound use and adherence to sterile technique, though there was a single postoperative infection, which may have resulted from other causes. The absence of a significant association between demographic factors, such as male gender, and recurrence may stem from a limited sample size, but it could also reflect the lack of a firm pathophysiological basis for such associations.

Limitations of this study include the lack of a published standard technique for the intraoperative ultrasound evaluation of CSDH. This is unsurprising due to the novelty of the technique. Operator dependence is also a known limitation of the ultrasound technique (23) and may result in variation in image quality and interpretation. In this study, Cohen’s kappa for interpreting the ultrasound images was 0.54, suggesting moderate inter-rater reliability, consistent with this known weakness. A standardised protocol may be necessary to reduce this variability. Although images were successfully obtained in all subjects, the narrow sonographic window provided by a single burr hole may limit complete interrogation of the hematoma and cortical surface. As this is a single-arm study, a true head-to-head comparison with CT imaging was not possible, and patient selection was not randomised but rather via convenience sampling, which may introduce selection bias. Surgical technique may also influence results, despite standardisation; notable differences, such as single vs. double burr hole, have not been shown to affect outcomes significantly. Multi-centre, prospective, randomised-controlled studies with standardised long-term follow-up will be necessary to validate and expand upon our findings.

## Conclusion

Intraoperative ultrasound can be used during burr-hole surgery to assess the removal of CSDH in real time. In this study, complete CSDH clearance on intraoperative ultrasound was associated with a lower recurrence rate, although the association was not statistically significant. Postoperative CT findings, including residual collections and pneumocranium, were also not significantly associated with recurrence. Significantly, these findings suggest that intraoperative ultrasound could be a valuable additional tool for assessing CSDH evacuation. However, its effectiveness in predicting recurrence remains unclear. Larger studies are required further to determine its clinical value in the management of CSDH.

## Figures and Tables

**Figure 1:**
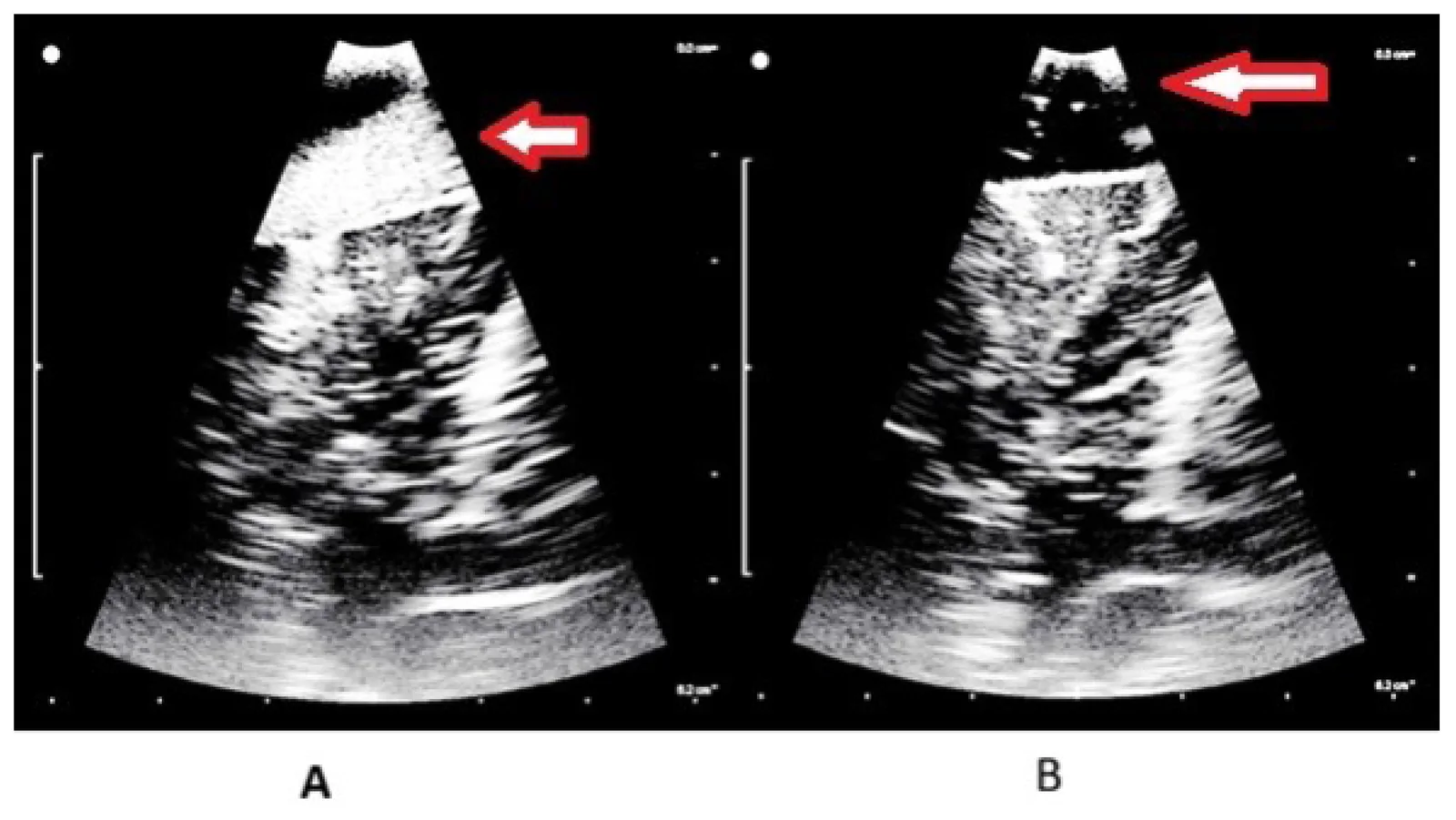
An example of intraoperative ultrasound: A) Pre-drainage demonstrating a double layer of hematoma; B) Post-drainage demonstrating a starry sky appearance signifying complete clearance, no membranes seen; Red closed arrows point towards hematoma.

**Figure 2:**
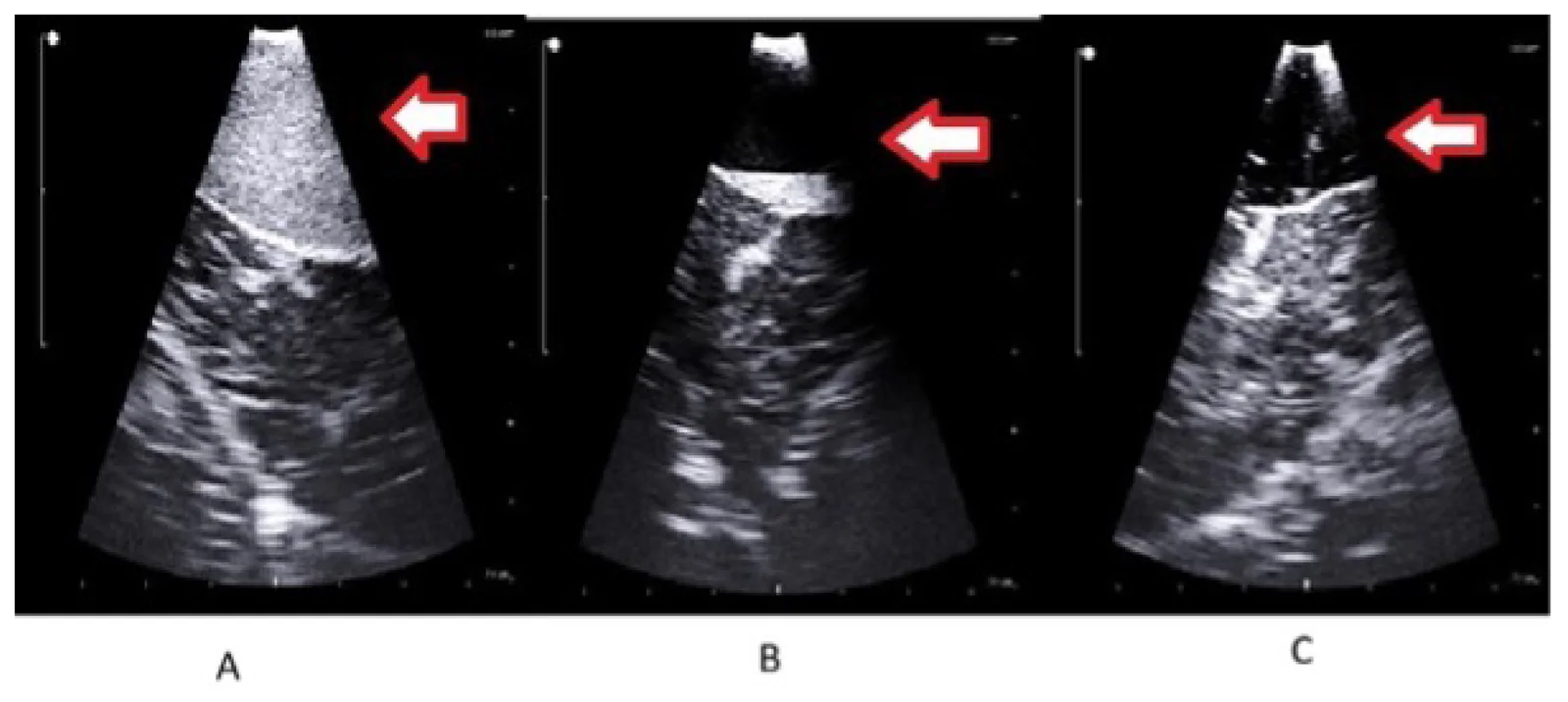
Another example of intraoperative ultrasound in another patient: A) Pre-drainage demonstrating a single homogeneous layer of hematoma; B) Post-drainage demonstrating residual deep hematoma; C) Further irrigation managed to clear hematoma, signifying complete clearance, no residual membranes seen; Arrows are pointing towards hematoma.

**Figure 3:**
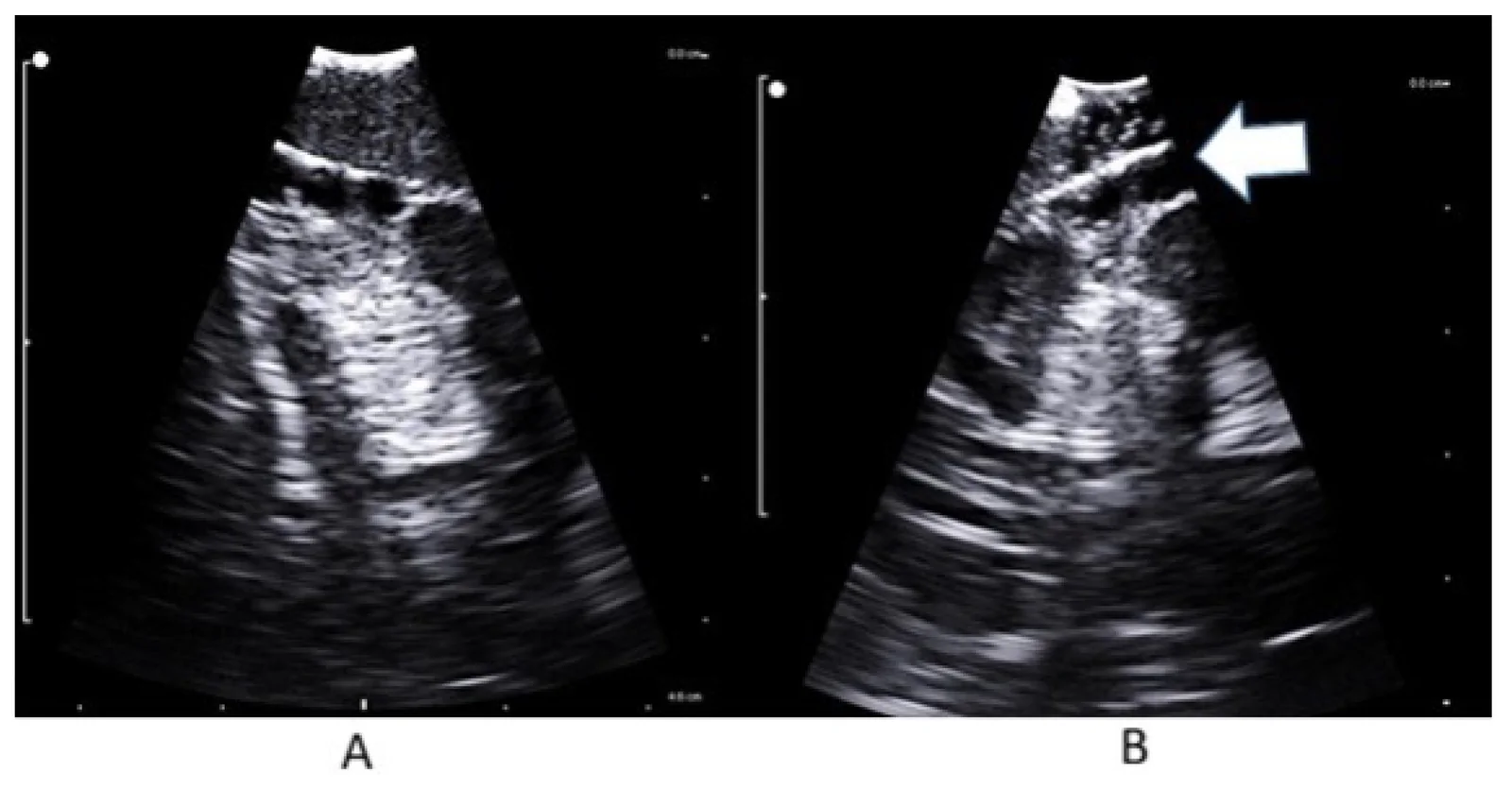
An example of residual following irrigation: A) Pre-drainage ultrasound demonstrating double layer of hematoma of differing densities – the deep hematoma showed trabeculations not seen on CT scan; B) Residual layer overlying cerebral cortex despite repeat irrigation; White arrow: No membrane manipulation was attempted here; Arrows are pointing at the hematoma layer.

**Table 1:** Demographics and preoperative characteristics.

Demographic data	Frequency (%)
Age (mean ± SD) (range)	69.3 ± 8.37 (53 to 87)
**Sex**	
Male	16 (64)
Female	9 (36)
Recent falls	14 (56)
Antiplatelet therapy	8 (32)
Anticoagulant therapy	5 (20)
**Co-morbidities**	
Hypertension	20 (80)
Ischaemic heart disease and cardiac disease	13 (52)
Previous stroke	8 (32)
Malignancy	3 (12)
Chronic kidney disease	1 (4)
**GCS score**	
13 to 15	17 (68)
8 to 12	8 (32)
**Symptoms**	
Motor weakness	14 (56)
Headaches	9 (36)
Altered sensorium	8 (32)
Seizures	1 (4)
Markwalder score 0 or 1 on admission	7 (28)
**Preoperative CT findings**	
Bilateral	10 (40), total 35 clots
Acute component	16 (64)
Average clot thickness (cm)	2.0 (1.3 to 3.0)
Average midline shift (cm)	0.67 (0.1 to 1.8) 0.92, bilateral excluded
Hematoma thickness	42
> 2 cm	
**Nakaguchi subtype**	
Homogenous	11 (31)
Laminated	5 (20)
Trabeculated	6 (26)
Separated	7 (23)

**Table 2:** Radiologic findings and postoperative outcomes.

Postoperative findings	Frequency (%)
**Intraoperative ultrasound**	
Cleared (starry sky)	12 (48)
Residual seen	13 (52)
**Postoperative CT**	
No residual haemorrhage or cleared	13 (52)
Pneumocranium	
Thin < 1 cm	14 (56)
Thick > 1 cm	11 (44)
Postoperative subdural collection (clot or otherwise) thickness	1.1 cm (0.2 to 2.2 cm) (45% decrease)
Postoperative midline shift	0.25 cm (0 to 0.6 cm) (73% decrease)
**Postop complications**	
Local complications	
Tension pneumocephalus	1 (4)
New acute subdural haemorrhage	2 (8)
Surgical site Infections and empyemas	1 (4)
Systemic complications	
Pneumonia	2 (8)
Cardiovascular	1 (4)
**Follow-up**	
Mean hospitalisation postop	5.96 days (3 to 17)
In hospital mortality	1 (4)
3-month mortality	2 (8)
3-month recurrence	4 (16)
Discharge mRS score (mean)	1.80
3-month mRS score (mean)	1.33

**Table 3:** Relationship between radiologic findings and clinical recurrence.

		Recurrence	No recurrence
Ultrasound	Clearance	0	12
	Residual	4	9
Postoperative CT	Clearance	2	11
	Residual	2	10

**Table 4:** Univariate analysis and odds ratio of relevant risk factors and operative findings.

Clinical variables	*P*-value (univariate)
Age	0.166
Gender	0.542
Diabetes Mellitus	0.265
Hypertension	0.166
Anticoagulant use	0.166
Antiplatelet use	0.188
Good Markwalder grade	0.527
Acute component	0.159
Thickness > 2 cm	0.458
Subtype	
Single/double burr-hole	0.532
Pneumocranium > 1 cm	0.108
	**Risk ratio of recurrence, OR (95% CI)**
Ultrasound - clearance	0.476 (0.304, 0.746)
CT imaging - clearance	0.750 (0.088, 6.388)
